# GPR30‐mediated estrogenic regulation of actin polymerization and spatial memory involves SRC‐1 and PI3K‐mTORC2 in the hippocampus of female mice

**DOI:** 10.1111/cns.13108

**Published:** 2019-02-03

**Authors:** Yuan‐Yuan Zhang, Meng‐Ying Liu, Zhi Liu, Ji‐Kai Zhao, Yan‐Gang Zhao, Li He, Wei Li, Ji‐Qiang Zhang

**Affiliations:** ^1^ Department of Neurobiology, Chongqing Key Laboratory of Neurobiology Third Military Medical University Chongqing China; ^2^ Department of Histology and Embryology Third Military Medical University Chongqing China; ^3^ School of Nursing Third Military Medical University Chongqing China

**Keywords:** actin polymerization, GPER, GPR30, membrane estrogen receptor, PI3K, rictor, spatial memory, steroid receptor coactivator‐1

## Abstract

**Aims:**

The G‐protein‐coupled estrogen receptor GPR30 (also referred to as GPER) has been implicated in the estrogenic regulation of hippocampal plasticity and spatial memory; however, the molecular mechanisms are largely unclear.

**Methods:**

In this study, we initially examined the levels of GPR30 in the hippocampus of postnatal, ovariectomy (OVX)‐ and letrozole (LET)‐treated female mice. Under G1, G15, and/or OVX treatment, the spatial memory, spine density, levels of ERα, ERβ, and SRC‐1, selected synaptic proteins, mTORC2 signals (Rictor and p‐AKT Ser473), and actin polymerization dynamics were subsequently evaluated. Furthermore, G1, G15, and/or E2 combined with SRC‐1 and/or PI3K inhibitors, actin cytoskeleton polymerization modulator JPK, and CytoD treatments were used to address the mechanisms that underlie GPR30 regulation in vitro. Finally, mTORC2 activator A‐443654 (A4) was used to explore the role of mTORC2 in GPR30 regulation of spatial memory.

**Results:**

The results showed that high levels of GPR30 were detected in the adult hippocampus and the levels were downregulated by OVX and LET. OVX induced an impairment of spatial memory, and changes in other parameters previously described were reversed by G1 and mimicked by G15. Furthermore, the E2 effects on SRC‐1 and mTORC2 signals, synaptic proteins, and actin polymerization were inhibited by G15, whereas G1 effects on these parameters were inhibited by the blockade of SRC‐1 or PI3K; the levels of synaptic proteins were regulated by JPK and CytoD. Importantly, G15‐induced actin depolymerization and spatial memory impairment were rescued by mTORC2 activation with A4.

**Conclusions:**

Taking together, these results demonstrated that decreased GPR30 induces actin depolymerization through SRC‐1 and PI3K/mTORC2 pathways and ultimately impairs learning and memory, indicating its potential role as a therapeutic target against hippocampus‐based, E2‐related memory impairments.

## 1 INTRODUCTION

Neurodegenerative diseases, such as Alzheimer's disease (AD), are the major threats in the global aging population without an effective cure.^1^ The hippocampus is the center for learning and memory and an important target of AD.^2^ Accumulated studies have demonstrated that hippocampal synaptic plasticity (including structural/morphological and functional plasticity) is the basis for memory formation and consolidation, which are profoundly affected by estrogens (particularly 17β estradiol, E2) both in vitro and in vivo.^3^, ^4^, ^5^, ^6^ It has been established that the structural plasticity of synapses (such as the spine density and synapse density) is a vital component of synaptic plasticity and behavior.^7^ Previous studies have shown that in the hippocampus, synapse density changes during the 4‐ to 5‐day estrous cycle of adult female rats,^8^ whereas further studies show that dendritic spines and the frequency of multiple synapse buttons are significantly regulated by E2 treatment in these rats.^9^, ^10^ Early studies have detected the expression of classic nuclear estrogen receptors α and β (ERα and ERβ; which have recently been localized in extranuclear components, such as the cell membrane and cytoplasm) in the hippocampus of both male and female rats^11^, ^12^ and they have been deeply involved in the estrogenic regulation of hippocampal synaptic plasticity, learning, and memory in both mice and rats.^13^, ^14^, ^15^ Moreover, our previous studies have shown mTORC2‐dependent regulation of actin polymerization that contributed to the effects of ERα and ERβ on the spatial learning of female mice^16^; however, the mechanisms that underlie estrogenic regulation of hippocampal morphological plasticity and ultimately learning and memory remain far from unclear.

The G‐protein‐coupled estrogen receptor GPR30 (also referred to as GPER or GPER1) was first reported by Carmeci et al^17^ using differential cDNA library screening from a breast cancer cell line; it was subsequently demonstrated to be the membrane estrogen receptor that mediates nongenomic estrogenic effects on target genes.^18^, ^19^ GPR30 immunoreactivities have been detected in the neuronal plasma membrane,^20^, ^21^ Golgi apparatus, and endoplasmic reticulum^21^, ^22^, ^23^ of female mice and/or rats. Limited studies using the GPR30 agonist G1 or the antagonist G15 have indicated a role of the GPR30 in spatial recognition memory,^24^, ^25^, ^26^, ^27^, ^28^ social recognition, object recognition, and object placement learning^28^ through distinct signaling pathways from ERα and/or ERβ^29^ that involve the regulation of hippocampal spine density^30^, ^31^ of female rats and mice; however, the detail regulation mechanisms remain to be elucidated.

Dendritic spines are specialized dendritic protrusions,^7^ and their structural plasticity is the basis of higher brain functions, such as learning and memory.^32^ The actin cytoskeleton is enriched in dendritic spines^33^; it exists as soluble globular monomers (G‐actin) and filamentous polymer (F‐actin) and dynamically shifts between G‐actin and F‐actin. F‐actin has been shown to be the main driving force and determinant of dendritic spine remodeling and synaptic plasticity,^34^, ^35^ which is controlled by the action of Profilin‐1 (responsible for the polymerization of G‐actin) and Cofilin (responsible for the depolymerization of F‐actin).^36^ Rictor is the rapamycin‐insensitive companion of mTORC2 and has been shown to regulate actin polymerization (F‐actin/G‐actin ratio) through the Tiam1‐RAC1‐phospho‐PAK and phospho‐Cofilin pathway and ultimately affect long‐term memory and the late phase of long‐term potentiation in male mice.^37^ GPR30 immunoreactivities have been detected in the dendritic spines and axon terminals of male and female mice,^23^ and in an attempt to investigate the differential role of classic nuclear estrogen receptors (ERα and ERβ) and membrane estrogen receptor GPR30 in the regulation of neuronal plasticity, we found that GPR30 inactivation induced a decrease in mTORC2 signals and synaptic proteins, as well as actin depolymerization, which were reversed by mTORC2 activation in female mice^38^; however, how GPR30 functions to regulate actin polymerization and the role that mTORC2 plays during this process are not completely understood.

To address these questions, in this study, we initially examined the profile of the GPR30 in the postnatal hippocampus of female mice and its changes in the hippocampus of ovariectomy (OVX)‐ or letrozole (LET)‐treated adult female mice. We subsequently examined the effects of the modulation of GPR30 activities on the behavior, spine density, actin polymerization (including the F‐actin/G‐actin ratio and the expression of classic nuclear estrogen receptors and their coactivator SRC‐1, selected synaptic proteins, and actin cytoskeleton remodeling proteins), and mTORC2 signals (Rictor, AKT, and p‐AKT). Furthermore, we explored the related mechanisms using antagonists against the SRC‐1 or PI3K pathway, as well as the actin polymerization stabilizer jasplakinolide (JPK) and disruptor cytochalasin D (CytoD) and the mTORC2 activator A4 in vivo and/or in vitro.

## 2 MATERIALS AND METHODS

### 2.1 Animals and drug administration

Different postnatal (from P0 to P56; P0 was defined as within the first 24 hours following birth) female C57BL/6 mice were obtained from the Experimental Animal Center of the Third Military Medical University and were used to investigate the hippocampal GPR30 profile at different postnatal stages. The adult female C57BL/6 mice (10‐12 weeks old, weighed 20‐22 g) were used for other experiments, such as OVX and LET/G1/G15 injection and behavior tests. The mice were group‐housed, and all animal‐related procedures were conducted in strict compliance with the Approved Institutional Animal Care and Use Protocols of this university (Reference No. SYXK‐PLA‐20120031). OVX was performed according to our previously reported procedures.^39^, ^40^ Briefly, mice were anesthetized with 4% chloral hydrate (100 mg/kg BW); the fur was clipped over the surgical area and scrubbed with Betadine and an ethanol swipe. The skin of the back was subsequently cut, and the bilateral ovaries were completely removed, followed by suture of the wound. The sham‐operated animals were used as control for OVX; in chemical injection experiments, the control mice were also injected with equal amount of DMSO when needed. For the non‐OVX mice (control), the vaginal smears were prepared and the cycling of all animals was examined with Tar purple staining; only the diestrous mice were used for further experiments. The behavior tests were conducted 7 day or 14 day after OVX, and then, the mice were sacrificed and used for Western blot analysis and Golgi staining.

The aromatase‐specific inhibitor LET (L6545, Sigma‐Aldrich Shanghai Trading Co Ltd, Shanghai, China) was dissolved in DMSO (D5879, Sigma‐Aldrich) and diluted with sterile saline solution prior to intraperitoneal injection (final concentration: 160 μg/kg BW). The selective GPR30 agonist G1 (10008933, Cayman Chemical Company, Michigan, USA) and the antagonist G15 (114673, Cayman Chemical) were dissolved in DMSO, respectively, and diluted with sterile saline. G1‐treated animals were subcutaneously injected with 100 μL G1 at the dose of 400 μg/kg BW G15‐treated animals were intraperitoneally injected with 100 μL G15 at the dose of 800 μg/kg BW The treatment time and dose for G1 and G15 were determined in a time‐ and dose‐dependent preliminary experiment based on their effects on the changes in selected hippocampal proteins (data not shown). For the LET treatment, the dose employed (160 μg/kg BW) was based on our previous studies that showed hippocampal SRC‐1 and actin polymerization were dose‐dependently regulated by LET.^41^ The mTORC2‐specific activator A4 (HY‐10425; Medchem Express, Shanghai, China)^37^ was prepared with DMSO and intraperitoneally injected with a dose of 2.5 mg/kg body weight as previous reports.^37^, ^38^ Animals in the DMSO and/or sham groups received an injection of an equal amount of vehicle (60% DMSO + 40% sterile saline solution) as indicated in each experiment. The injection was conducted every morning at 08:30‐09:30 and lasted for 1 week. For better understanding the role of G1 activation on sham animals, two additional groups, namely sham and sham + G1, were set and Western blot on selected molecules was conducted.

### 2.2 Morris water maze behavior test

Behavior test was conducted 1 or 2 weeks after OVX of chemical injection. The learning and memory test was performed using the classic Morris water maze, which was conducted from 14:00 in the afternoon. The mice (n = 10 for each group) were adapted to the environment and pretrained to climb the escape platform in a closed alley on the day prior to the behavior test; the mice subsequently underwent a 6‐day testing phase with a submerged platform (10 × 10 cm) in a circular black pool (diameter: 120 cm) filled with white opaque water. The maze was divided into four quadrants: the left quadrant (L), the right quadrant (R), the target quadrant (T), and the opposite quadrant (O). During the 5‐day training period, the platform location and the starting point in every quadrant were maintained constant. The training consisted of four trials in which the mice had a maximum of 60 seconds to find the platform, followed by a 10‐second rest time on the platform after each trial. The mice were allowed to rest in a warmed cage for at least 20 minutes between the trials throughout the experiment. The spatial probing test was conducted on the 6th day, for which the platform was removed, and the trial duration remained 60 seconds. The time to find the platform and the time spent in the target zone were automatically monitored and recorded by a tracking system connected to an image analyzer (HVS Image, Hampton, UK). In addition, the swim speed and distance traveled of each mouse were recorded with the same tracking system. After behavior test, mice were either subjected to Golgi staining or Western blot analysis.

### 2.3 Golgi‐Cox staining for dendritic spines

After behavior test, one part of the mice (n = 3) were used for Golgi‐Cox staining, and this staining was used to evaluate the changes in the dendritic spines of the hippocampal CA1 area following the guide of the FD Rapid GolgiStain™ Kit (FD Neurotechnologies, Ellicott City, USA). The mice were deeply anaesthetized with 100 mg/kg sodium pentobarbital, and the brains were rapidly dissected and rinsed with distilled water. The brains were subsequently immersed in the impregnation solution, which was prepared by mixing equal volumes of Solutions A and B at room temperature in the darkroom with a change in the solution on the following day. After 2 weeks, the tissue was transferred to Solution C and stored at room temperature in the darkroom. The solution was changed on the next day and subsequently maintained for 1 week. The tissues were embedded with agarose and subsequently cut into 200‐μm‐thick coronal sections with a vibratome (Microslicer DTK‐600; Dosaka EM, Tokyo, Japan). Each section was mounted on gelatin‐coated microscope slides. All sections were dried at room temperature in the darkroom for 3 days. The sections were rinsed in distilled water (two times, 4 minutes each) and were subsequently placed in a chromogenic mixture (10 mL Solution D, 10 mL Solution E, and 20 mL double distilled water) for 10 minutes. After additional washes with distilled water, the sections were dehydrated with gradient alcohol solution (50%, 75%, and 95% ethanol for 4 minutes each and 4 minutes in 100% ethanol for four times). Finally, the sections were cleared in xylene and mounted.

The images were recorded with a Leica DP70 digital camera equipped with an Olympus microscope. Usually, 18‐20 neurons were recorded, and the number of dendritic spines was counted along the secondary branching of dendrites with Image‐Pro Plus software following the user guide, and the mean number of every 10 μm of dendritic length was automatically calculated with the same software.

### 2.4 F‐actin/G‐actin ratio measurement

Actin polymerization, which is based on F‐actin/G‐actin dynamics, has been shown to play a crucial role in the formation of memory.^37^ After behavior test, protein of the hippocampus from three mice was extracted and the F‐actin to G‐actin ratio was determined by Western blot as previously described.^37^, ^41^ Briefly, the hippocampus was isolated and homogenized in cold lysis buffer (10 mmol/L K2HPO_4_, 100 mmol/L NaF, 50 mmol/L KCl, 2 mmol/L MgCl_2_, 1 mmol/L EGTA, 0.2 mmol/L dithiothreitol, 0.5% Triton X‐100, 1 mol/L sucrose, pH 7.0) and then centrifuged at 15 000 *g* for 30 minutes. G‐actin (soluble actin) was measured with a rabbit polyclonal anti‐actin antibody in the supernatant. To measure F‐actin, the pellets were resuspended in an equal volume of lysis buffer (1.5 mmol/L guanidine hydrochloride, 1 mmol/L sodium acetate, 1 mmol/L CaCl_2_, 1 mmol/L ATP, 20 mmol/L Tris‐HCl, pH 7.5) and incubated on ice for 1 hour to depolymerize F‐actin with gentle mixing every 15 minutes. The samples were centrifuged at 15 000 *g* for 30 minutes, and this supernatant was used to measure actin with the same antibody (as a reflection of insoluble F‐actin). Samples from the supernatant (G‐actin) and pellet (F‐actin) fractions were proportionally loaded and analyzed with Western blot.

### 2.5 Cell culture and drug treatment

Cells of the embryonic mouse hippocampal cell line mHippoE‐14 (CELLutions Biosystems Inc, Canada), which have been shown to express all ERs, SRC‐1, GluR1, and PSD95, as well as several other factors,^42^ were cultured in DMEM with 10% fetal bovine serum (Gibco, 10099‐141, Shanghai, China), 25 mmol/L glucose, and 1% penicillin/streptomycin and were maintained at 37°C with 5% CO_2_ in a humidified atmosphere. For E2 treatment, cells were starved for 2 days with serum‐free medium. On the third day, 10^−6^ mol/L E2 (ab120657, Abcam Shanghai Trading Co Ltd, Shanghai, China) prepared with DMSO was added to the serum‐free medium and cultured for an additional 2 day. Different drug treatments were subsequently conducted. These drugs were dissolved in DMSO, respectively, and diluted to the final concentration prior to use. The final concentrations were 10^−6^ mol/L for G1 or G15, 10^−8^ mol/L for the SRC‐1^43^‐specific inhibitor Bufalin (sc‐200136, Santa Cruz) or the PI3K‐specific inhibitor Wortmannin^44^ (S1952, Beyotime, Shanghai, China), and 2 × 10^−7^ mol/L for the actin cytoskeleton polymerization stabilizer JPK^37^ (102396‐24‐7, Santa Cruz) or the disruptor CytoD^37^ (22144‐77‐0, Santa Cruz). The medium was replaced with fresh DMEM that contained the drugs and incubated for an additional 48 hours; these experiments were repeated at least three times. The dose for the chemicals was based on previous reports as indicated above or our preliminary assessments of their dose‐dependent regulation on selected proteins.

### 2.6 Western blot analysis

Western blot analysis was conducted according to our previous description.^45^ In general, after OVX or behavior test, hippocampi from mice (n = 3) were dissected and the hippocampal proteins were extracted using a Protein Extract Kit (P0027, Beyotime Biotech), and the protein concentration was determined using a BCA Assay Kit (P0010, Beyotime Biotech). Membranes were blocked with 5% fresh‐prepared milk‐TBST for 2 hour at room temperature and were subsequently incubated with the individual diluted primary antibodies at 4°C overnight. After TBST washes, the membranes were incubated with HRP‐conjugated goat anti‐rabbit secondary antibody (1:2000, ZB‐2301, Zhongshan Biotech) or goat anti‐mouse secondary antibody (1:2000, ZB‐2305, Zhongshan Biotech, Beijing, China) for 1.5 hour at 37°C, respectively. The blots were visualized with chemiluminescent HRP Substrate (WBKLS0100, Merck Millipore) for 5 min with Western Lightning‐ECL (Bio‐Rad, USA). The experiments were repeated at least three times. For the measurement of phospho‐AKT (Ser473) (p‐AKT) and total AKT, p‐AKT was first examined with the protocol as previously described; the membranes were subsequently washed with a specific stripping buffer (P0023A, Beyotime Biotech), blocked with 5% fresh‐prepared milk, incubated with the primary antibodies against AKT, and then continued to the next step.

The optical density for each band was measured using Quantity One software (Bio‐Rad) and was normalized to that of β‐actin or AKT (for p‐AKT). A blank control (without the primary antibody) was used to determine the specificity of the primary antibodies, and a prestained protein marker was employed to localize the specific band of the primary antibodies. The experiments were repeated at least three times.

The primary antibodies of Western blot were as follows: rabbit anti‐GPR30 (1:50; sc‐48525‐R, Santa Cruz Biotech, Dallas, USA), rabbit polyclonal anti‐ERα (1:100; sc‐542, Santa Cruz), rabbit polyclonal anti‐ERβ (1:100; sc‐8974, Santa Cruz), rabbit anti‐SRC‐1 (1:600; sc‐8995, Santa Cruz), rabbit mAb‐PSD95 (1:1000; 3409, Cell Signaling), rabbit mAb‐GluR1 (1:1000; 04‐855, Merck Millipore), rabbit mAb‐Rictor (1:500; 2140, Cell Signaling), rabbit mAb‐phospho‐AKT (Ser473) (1:2000; 9271, Cell Signaling), rabbit mAb‐AKT (1:2000; 9272, Cell Signaling), rabbit mAb‐Cofilin (1:300; 3312, Cell Signaling), rabbit polyclonal anti‐Profilin‐1 (1:4000; GTX63456, GeneTex), rabbit polyclonal anti‐actin (1:1000; GTX61452, GeneTex), and mouse mAb‐β‐actin (1:1,000; AA128, Beyotime Biotech).

### 2.7 Statistical analysis

All results are shown as the mean ± SEM SPSS version 13.0 (IBM; Chicago, IL) was used for the statistical analysis. The measurements were analyzed using independent samples *t* tests for two groups and one‐way ANOVA with the LSD test for three and more groups. For the water maze test, repeated measures of two‐way ANOVA with the LSD test were used for the learning analysis and one‐way ANOVA with the LSD test was used for the memory test. The statistical significance was defined as *P* < 0.05.

## 3 RESULTS

### 3.1 GPR30 increased with postnatal development and decreased with estrogenic deprivation

**Figure 1:**
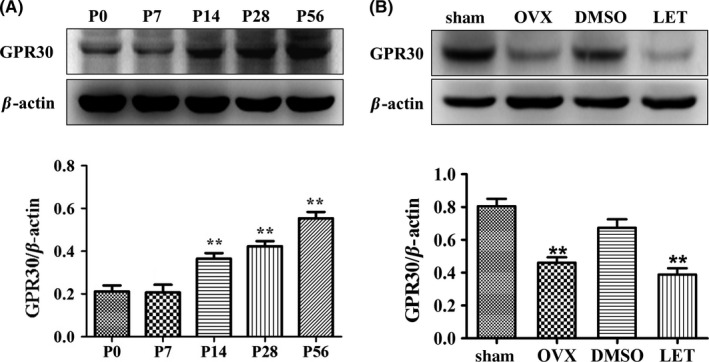
Hippocampal GPR30 was regulated by postnatal development and estrogenic deprivation. The P56 and sham animals were in diestrous cycling (evidenced by vaginal smear). The sham animals were used as the control for OVX, and the diestrous mice injected with DMSO were used as the control for LET injection. A, Hippocampal GPR30 increased with postnatal development, and the highest levels were detected at P56. The experiments were repeated three times, and data are shown as the mean ± SEM **P < 0.01 compared with P0 (one‐way ANOVA, LSD test). B, Hippocampal GPR30 was significantly decreased by OVX or LET. The experiments were repeated three times, and data are shown as the mean ± SEM OVX: ovariectomy. LET, letrozole. **P < 0.01 compared with corresponding control (one‐way ANOVA, LSD test)

We initially examined the expression of GPR30 in the postnatal hippocampus of female mice from P0 to P56. As shown in Figure 1A, Western blot and one‐way ANOVA analysis indicated that there were general differences among P0‐P56 (*F*(4,10) = 27.089, *P* < 0.01). Low levels of GPR30 were identified at P0 and P7, and there was no significant difference between P0 and P7 (*P* > 0.05). The level subsequently increased to a higher level at P14, and there was no significant difference between P14 and P28 (*P* > 0.05). The levels of GPR30 reached the highest levels at P56 (*P* < 0.05 compared with P28 and *P* < 0.01 compared with P0, P7, or P14).

We subsequently examined whether the expression of hippocampal GPR30 was affected by the deprivation of circulating estrogens using OVX or E2 synthesis using the administration of the aromatase‐specific inhibitor LET. Western blot and one‐way ANOVA analysis indicated that 1 week after treatment, there were significant differences among the sham, OVX, DMSO, and LET (*F*(3, 20) = 7.791, *P* < 0.01). In the adult hippocampus of the female mice, the expression of GPR30 was significantly decreased by the 7‐day OVX treatment (38% decrease; *P* < 0.01 compared with the sham animals) or 160 μg/kg LET (43% decrease; *P* < 0.01 compared with the DMSO‐treated control animals) as shown in Figure 1B.

### 3.2 GPR30 activation reversed OVX‐induced spatial memory impairment, CA1 dendritic spine loss, and actin depolymerization

**Figure 2:**
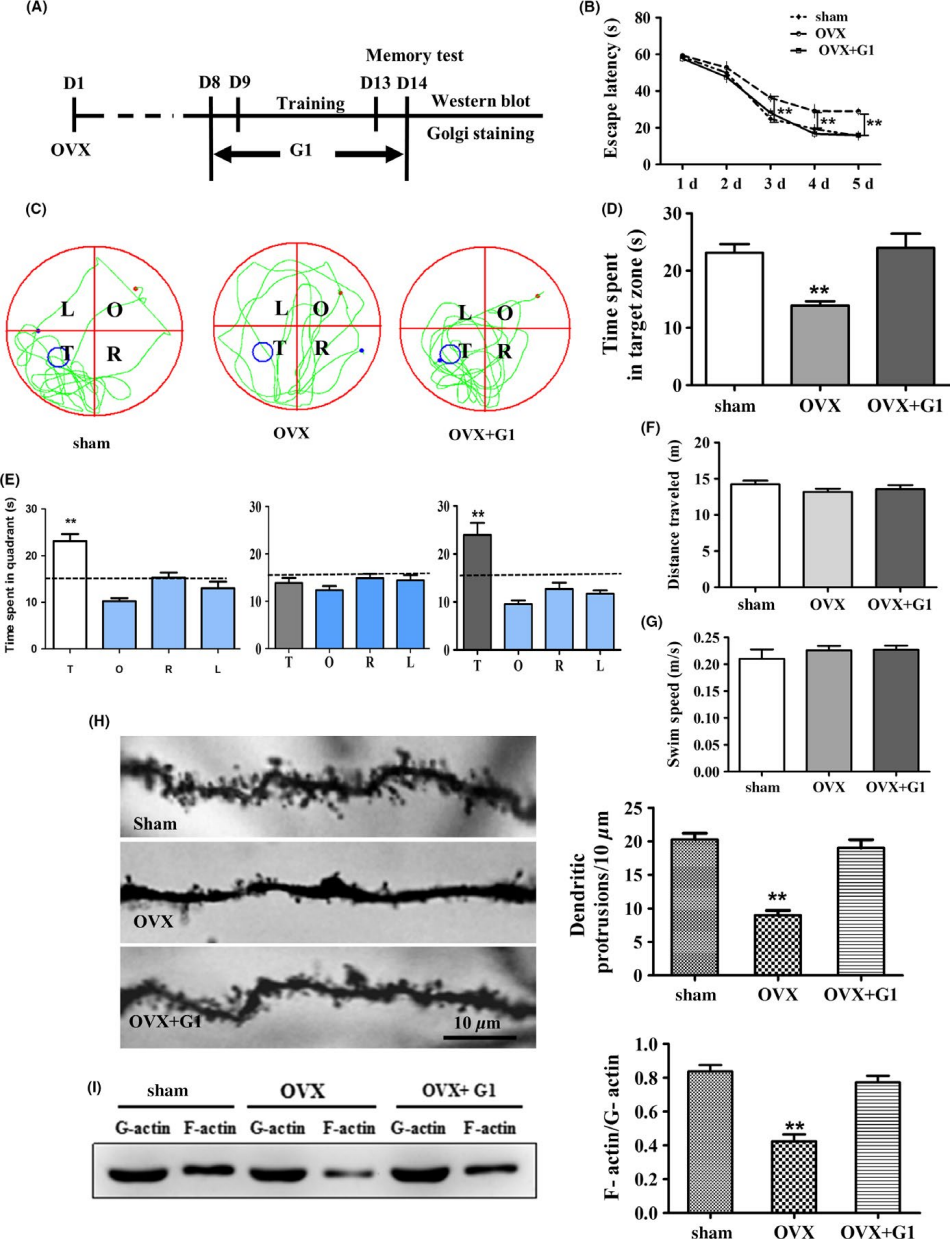
Activation of GPR30 with G1 restored OVX‐induced impairment of behavior, decrease in dendritic spine density, and depolymerization of actin. The sham animals were in diestrous cycling (evidenced by vaginal smear), were injected with an equal amount of DMSO as in the G1‐treated mice, and were used as the control. A, Schematic illustration of animal training, G1 administration, and behavior test and subsequent examines. B, OVX induced a significantly longer escape latency from day 3 to day 5, which was restored by G1. C, The swimming tracks of mice in each quadrant. D‐E, The time spent in the target quadrant of animals. F‐G, The total distance traveled and swimming speed. H, The changes in CA1 dendritic spine density. I, The changes in F‐actin/G‐actin ratio. L, left quadrant; R, right quadrant; T, target quadrant; O, opposite quadrant. Bar = 10 μm. Data are shown as the mean ± SEM **P < 0.01 compared with other groups (repeated measures of two‐way ANOVA, LSD test for water maze test and one‐way ANOVA, LSD test for spine density and F‐actin/G‐actin)

As the levels of GPR30 were decreased after OVX or LET injection, we subsequently aimed to explore the role of GPR30 in the estrogenic regulation of hippocampus‐dependent spatial learning and memory, as well as synaptic plasticity. G1 is the first identified GPR30‐specific agonist.^46^ To examine the role of the GPR30 in estrogenic regulation on learning and memory, adult female mice were castrated, and 7 day later, they were injected with G1 and the sham animals were injected with the same volume of DMSO under the strategy shown in Figure 2A. The Morris water maze test and two‐way ANOVA analysis showed that during the 5‐day training phase, the escape latency was significantly affected by both day (*F* = 172.196, *P* < 0.01) and treatment (*F* = 11.314, *P* < 0.01); there was also a detectable day × treatment interaction (*F* = 19.101, *P* < 0.01). Furthermore, the OVX animals showed significantly longer escape latency than the sham animals; this deficiency was substantially reversed by G1 injection. At day 6, the memory test day, one‐way ANOVA indicated there were significant differences among the Sham, OVX, and OVX + G1 (*F*(2,15) = 10.319, *P* < 0.01); the OVX animals spent a shorter time in the target zone than the sham animals (39% decrease; *P* < 0.01), whereas the G1 animals showed a similar time spent in the target zone as detected in the sham animals (*P* < 0.01 compared with the OVX animals) as shown in Figure 2B‐E. However, the total swimming distance and swimming speed were not significantly different among the three groups as indicated in Figure 2F‐G (Figure 2F: *F*(2,15) = 1.145, *P* > 0.05; Figure 2G: *F*(2,15) = 0.621, *P* > 0.05).

We subsequently examined the changes in the CA1 dendritic spine density using Golgi‐Cox staining. One‐way ANOVA showed that there were significant differences among the Sham, OVX, and CA1. OVX substantially decreased the CA1 spine density compared with the sham animals (*P* < 0.01 compared with sham). However, this decrease was significantly reversed by G1 treatment (Figure 2H; *P* < 0.01 compared with OVX). For the F‐actin/G‐actin ratio, Western blot analysis indicated there were significant differences among these three groups (*F*(2,15) = 32.465, *P* < 0.01); OVX induced a significant decrease in F‐actin/G‐actin (*P* < 0.01 compared with sham animals), and this effect was reversed by G1 treatment (*P* < 0.01 compared with OVX animals) to the sham levels as shown in Figure 2I.

### 3.3 GPR30 activation reversed OVX‐induced changes in classic nuclear ERs and SRC‐1, synaptic proteins, mTORC2 signals, and actin remodeling proteins in vivo

**Figure 3:**
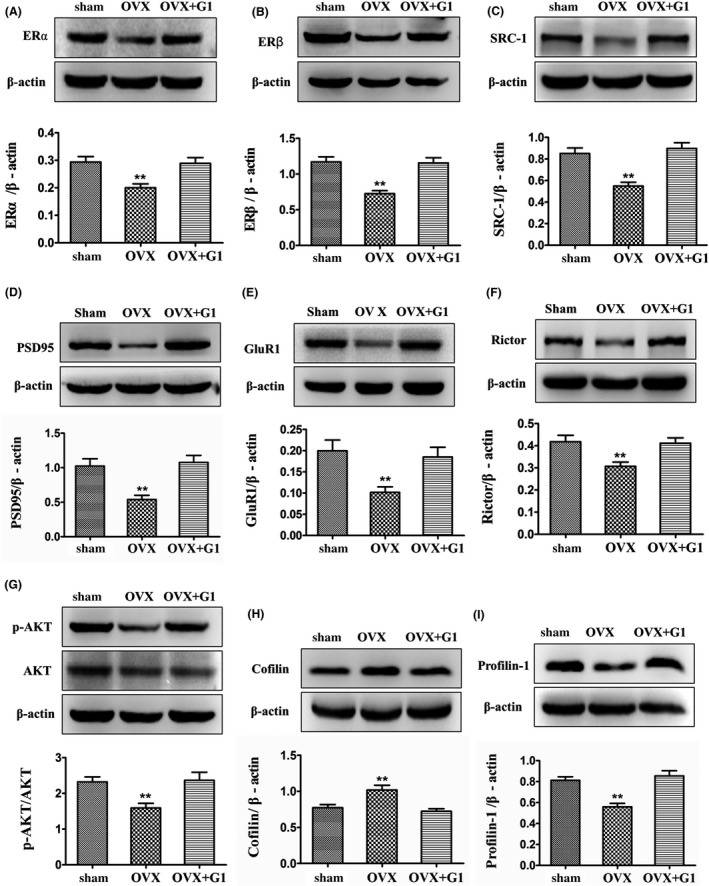
Activation of GPR30 with G1 restored OVX‐induced changes in classic nuclear ERs and their coactivator SRC‐1, synaptic proteins, mTORC2 signals, and actin polymerization remodeling proteins. The sham animals were in diestrous cycling (evidenced by vaginal smear) and were injected with an equal amount of DMSO as in the G1‐treated mice. A‐B, Estrogen receptors α and β. C, SRC‐1. D‐E, Synaptic proteins (GluR1 and PSD95). F‐G, mTORC2 signals (Rictor and p‐AKT). H‐I, actin polymerization remodeling proteins. The experiments were repeated three times, and data are shown as the mean ± SEM **P < 0.01 compared with other groups (one‐way ANOVA, LSD test)

Based on the observations that OVX and G1 significantly affected learning and memory, as well as the CA1 dendritic spine density, we subsequently examined the effects of these treatments on the expression of specific proteins shown to be responsible for actin polymerization remodeling and synaptic transmission. The classic nuclear estrogen receptors ERα and ERβ have been implicated in the regulation of learning and memory,^13^, ^15^, ^47^ and SRC‐1 has been shown to be involved in the LET regulation of hippocampal PSD95^48^ and actin polymerization.^41^ Western blot and one‐way ANOVA analysis indicated that the levels of ERα, ERβ, and SRC‐1 in the hippocampus of the female mice were significantly different among the Sham, OVX, and OVX + G1 (*F*(2,15) = 7.895, *P* < 0.01 for ERα; *F*(2,15) = 10.128, *P* < 0.01 for ERβ; *F*(2,15) = 10.575, *P* < 0.01 for SRC‐1). Further analysis indicated these proteins were downregulated by OVX compared with the sham animals (by 32%, 34%, and 33%, respectively; *P* < 0.01). However, this inhibition was significantly reversed by G1 treatment compared with the OVX animals (*P* < 0.01) and reached the sham level as shown in Figure 3A‐C.

PSD95 and GluR1 are important postsynaptic proteins of excitatory synapses that function to regulate neuronal excitability and signal transmission. One‐way ANOVA analysis indicated there were significant differences among the Sham, OVX, and OVX + G1 (*F*(2,15) = 10.366, *P* < 0.01 for PSD95 and *F*(2,15) = 6.265, *P* < 0.05 for GluR1). In the hippocampus of the OVX animals, the levels of PSD95 and GluR1 were significantly decreased by 47% and 49%, respectively, compared with the sham animals (*P* < 0.01). However, in the hippocampus of the OVX + G1 animals, the levels of these proteins were significantly increased compared with the OVX animals (*P* < 0.01) and reached the sham level. The results are shown in Figure 3D‐E.

Rictor and its downstream p‐AKT have been shown to regulate actin polymerization.^37^ Profilin‐1 and Cofilin have been demonstrated to directly control actin cytoskeleton polymerization.^36^ For Rictor, p‐AKT, Profilin‐1, and Cofilin, one‐way ANOVA analysis showed that there were significant differences among the Sham, OVX, and OVX + G1 (*F*(2,15) = 6.709, *P* < 0.01 for Rictor; *F*(2,15) = 6.511, *P* < 0.01 for p‐AKT; *F*(2,15) = 14.923, *P* < 0.01 for Profilin‐1; *F*(2,15) = 10.081, *P* < 0.01 for Cofilin). In the hippocampus of the OVX animals, the levels of Rictor, p‐AKT, and Profilin‐1 were significantly decreased by 26%, 32%, and 30%, respectively, compared with the sham animals (*P* < 0.01); in contrast, the levels of Cofilin were significantly increased by 32% compared with the sham animals (*P* < 0.01). However, in the hippocampus of the OVX + G1 animals, the levels of these proteins were significantly reversed to the sham level compared with the OVX animals (*P* < 0.01). The results are shown in Figure 3F‐I.

Finally, for the activating effects of G1 on selected hippocampal chemicals in Sham animals, *t* test revealed that all the examined proteins, except p‐AKT (*t* = 2.748, *P* > 0.05, compared to sham), were significantly up‐regulated by G1 treatment (*t* = 5.089, *P* < 0.01 for ERα; *t* = 7.807, *P* < 0.01 for SRC‐1; *t* = 3.988, *P* < 0.05 for PSD95; *t* = 3.269, *P* < 0.05 for GluR1; *t* = 4.951, *P* < 0.01 for Rictor; and *t* = 2.913, *P* < 0.05 for Profilin‐1). These results showed that G1 is effective for adult females under normal physiological conditions (Figure S1).

### 3.4 GPR30 inactivation impaired spatial memory, downregulated CA1 spine density, and induced actin depolymerization

**Figure 4:**
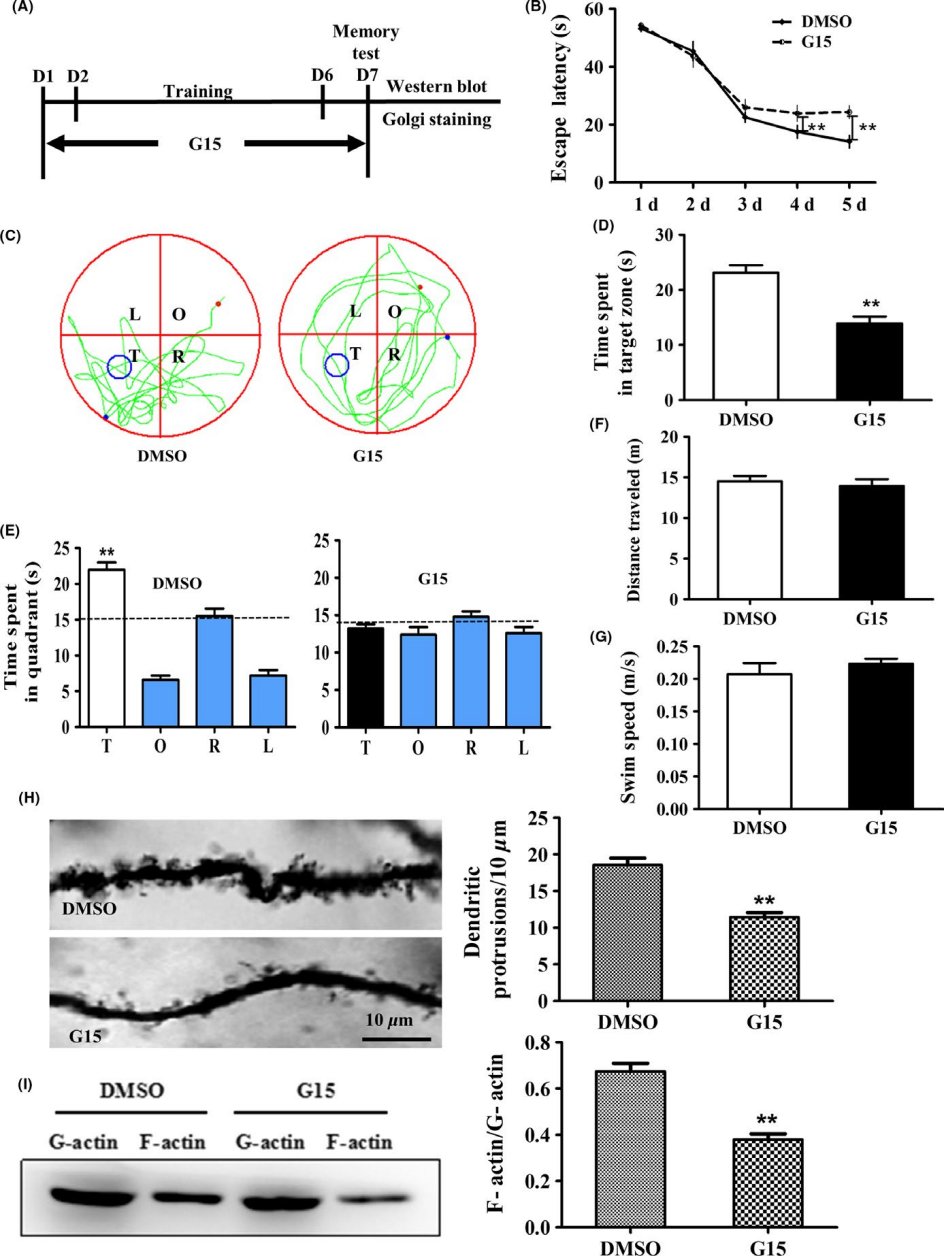
Inactivation of GPR30 with G15‐induced impairment of behavior, decreases in dendritic spine density, and depolymerization of actin. The adult diestrous (evidenced by vaginal smear) mice were used for experiments; diestrous mice were injected with an equal amount of DMSO as in the G15‐treated mice and were used as the control for G15 treatment. A, Schematic illustration of animal training, G15 administration, and behavior test and subsequent examines. B, G15 induced significantly longer escape latency from day 4 to day 5. C, The swimming tracks of mice in each quadrant. D‐E, The time spent in the target quadrant of animals. F‐G, The total distance traveled and swimming speed. H, The changes in CA1 dendritic spine density. I, The changes in F‐actin/G‐actin ratio. L, left quadrant; R, right quadrant; T, target quadrant; O, opposite quadrant. Bar = 10 μm. Data are shown as the mean ± SEM **P < 0.01 compared with DMSO (repeated measures of two‐way ANOVA, LSD test for water maze test and one‐way ANOVA, LSD test for spine density and F‐actin/G‐actin)

G15 is the specific antagonist against the GPR30.^49^ We treated the mice with G15 (20.0 μg/mouse) for 7 day and subsequently conducted behavioral testing using the Morris water maze test under the strategy indicated in Figure 4A. For the G15 treatment, two‐way ANOVA indicated that the escape latency was significantly affected by both day (*F* = 76.833, *P* < 0.01) and treatment (*F* = 7.306, *P* < 0.05); however, there was no day × treatment interaction (*F* = 1.663, *P* > 0.05). The total swimming distance and the swimming speed were not significantly different (*P* > 0.05) compared with the DMSO‐treated controls as shown in Figure 4B‐G.

For the changes in the CA1 dendritic spine density, Golgi‐Cox staining indicated that the spine density was significantly decreased by 37% after G15 treatment compared with the DMSO control as shown in Figure 4H (*t* = 6.724, *P* < 0.01). Moreover, the F‐actin/G‐actin ratio was significantly decreased by 43%, as demonstrated by Western blot, compared with DMSO as shown in Figure 4I (*t* = 6.287, *P* < 0.01).

### 3.5 GPR30 inactivation regulated classic nuclear ERs and SRC‐1, synaptic proteins, mTORC2 signals, and actin remodeling proteins in vivo

**Figure 5:**
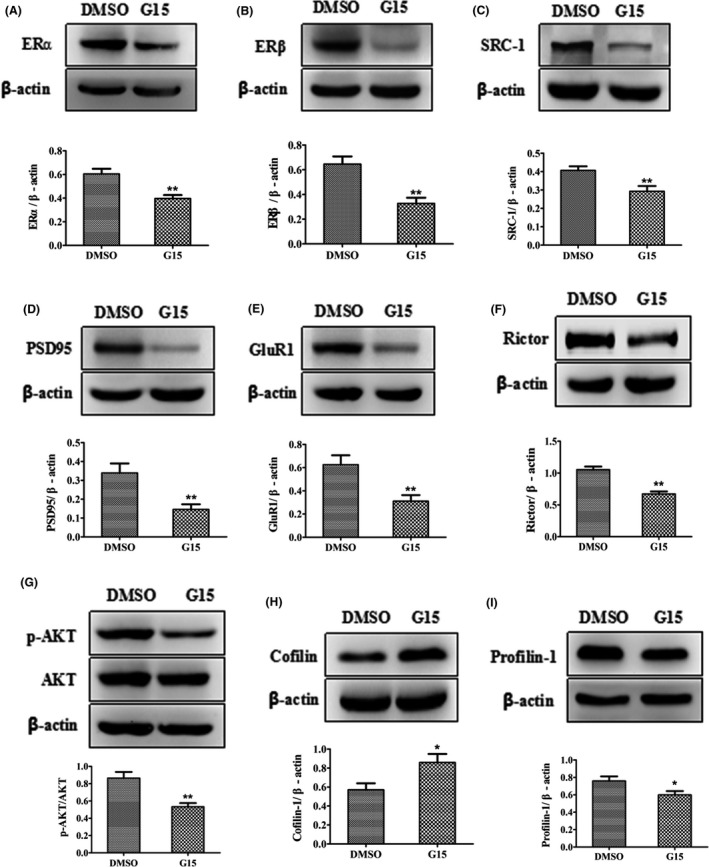
Inactivation of GPR30 with G15‐induced changes in ERα, ERβ, and their coactivator SRC‐1, synaptic proteins, mTORC2 signals, and actin polymerization remodeling proteins. The adult diestrous (evidenced by vaginal smear) mice were used for experiments; diestrous mice were injected with an equal amount of DMSO as in the G15‐treated mice and were used as the control for G15 treatment. A‐B, Estrogen receptors α and β. C, SRC‐1. D‐E, synaptic proteins (GluR1 and PSD95). F‐G, mTORC2 signals (Rictor and p‐AKT). H‐I, actin polymerization remodeling proteins. The experiments were repeated three times, and data are shown as the mean ± SEM *P < 0.05, **P < 0.01 compared with DMSO (independent samples *t* tests)

To further explore the related mechanisms under pharmacological inactivation of the GPR30 on spatial learning and spine density, we examined several actin polymerization remodeling signals. Western blot analysis showed that the levels of ERα were significantly decreased by 34% after G15 treatment (*t* = 3.992, *P* < 0.01 compared with DMSO). For ERβ, the levels were decreased by 50% (*t* = 4.100, *P* < 0.01 compared with DMSO). FOR SRC‐1, the levels were significantly decreased by 28% after G15 treatment (*t* = 3.118, *P* < 0.01 compared with DMSO). These results are shown in Figure 5A‐C. For the hippocampal postsynaptic proteins GluR1 and PSD95, the levels were significantly decreased by 52% and 57%, respectively, after G15 treatment compared with the DMSO‐treated animals as shown in Figure 5D‐E (Figure 5D; *t* = 3.372, *P* < 0.01 for PSD95; Figure 5E: *t* = 3.283, *P* < 0.01 for GluR1). Moreover, the levels of Rictor and p‐AKT were significantly decreased by 36% and 38%, respectively, after G15 treatment compared with the DMSO‐treated mice (Figure 5F; *t* = 6.192, *P* < 0.01 for Rictor; Figure 5G: *t* = 3.965, *P* < 0.01 for p‐AKT). Furthermore, the levels of Cofilin were significantly increased by 52% after G15 treatment compared with the DMSO‐treated animals (Figure 5H; *t *= −2.545, *P* < 0.01 for Cofilin). Profilin‐1 was significantly decreased by 25% after G15 treatment compared with the DMSO‐treated mice (Figure 5I; *t* = 2.465, *P* < 0.01 for Profilin‐1).

### 3.6 GPR30 inactivation inhibited E2‐induced changes in SRC‐1, synaptic proteins, mTORC2 signals, and actin remodeling proteins in vitro

**Figure 6:**
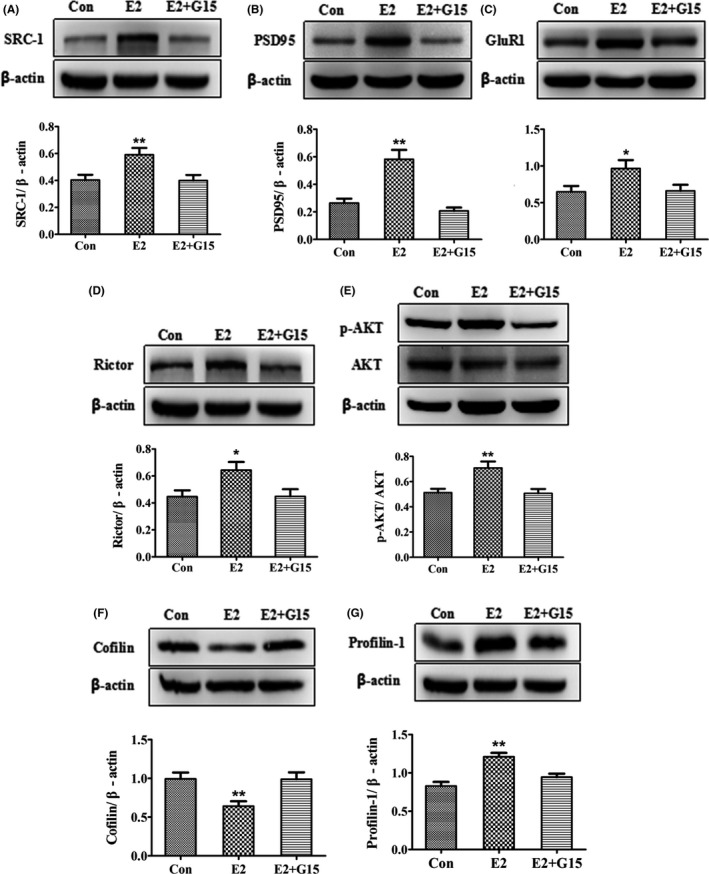
E2‐induced changes in SRC‐1, synaptic proteins, mTORC2 signals, and actin polymerization remodeling proteins were inhibited by inactivation of GPR30 with G15 in vitro. A, SRC‐1. B‐C, Synaptic proteins (GluR1 and PSD95). D‐E, mTORC2 signals (Rictor and p‐AKT). F‐G, actin polymerization remodeling proteins. Con, control (DMSO treated). The experiments were repeated three times, and data are shown as the mean ± SEM *P < 0.05, **P < 0.01 compared with other groups (one‐way ANOVA, LSD test)

The embryonic mouse hippocampal cell line mHippoE‐14 has been shown to express GPR30 in a previous study.^50^ To verify the in vivo effects of the GPR30 on the regulation of actin cytoskeleton remodeling proteins and synaptic proteins, we assessed whether the effects of E2 on the expression of these proteins could be inhibited by G15 treatment. Using mHippoE‐14 cells, we determined that for SRC‐1, mTORC2 signals, actin remodeling proteins, and synaptic proteins, there were significant differences among the control, E2, and E2+G15 (*F*(2,15) = 6.252, *P* < 0.05 for SRC‐1; *F*(2,15) = 22.615, *P* < 0.01 for PSD95; *F*(2,15) = 3.774, *P* < 0.05 for GluR1; *F*(2,15) = 4.409, *P* < 0.05 for Rictor; *F*(2,15) = 8.096, *P* < 0.01 for p‐AKT; *F*(2,15) = 6.675, *P* < 0.01 for Cofilin; and *F*(2,15) = 15.588, *P* < 0.01 for Profilin‐1). The expression of SRC‐1 was significantly increased by 48% after E2 treatment (*P* < 0.01 compared with control); however, this effect was significantly inhibited by a combined G15 treatment as shown in Figure 6A. For synaptic proteins, the levels of PSD95 and GluR1 increased by 59% (*P* < 0.01) and 49% (*P* < 0.05), respectively, after E2 treatment; moreover, the E2‐induced regulation of these proteins was inhibited to the control levels by combined G15 treatment as shown in Figure 6B‐C. For mTORC2 signals and actin remodeling proteins, the expressions of Rictor and p‐AKT were increased by 45% and 39%, respectively, after E2 treatment (*P* < 0.01 compared with control) and inhibited by G15 treatment as shown in Figure 6D‐E. However, the expression of Cofilin was decreased by 36% after E2 treatment (*P* < 0.01 compared with DMSO) and increased to the DMSO level after G15 treatment (Figure 6F). Profilin‐1 was increased by 38% after E2 treatment (*P* < 0.01 compared with control) and inhibited by G15 treatment as shown in Figure 6G.

### 3.7 GPR30 activation‐induced changes in actin remodeling proteins and synaptic proteins were inhibited by blockade of SRC‐1 and PI3K in vitro

**Figure 7:**
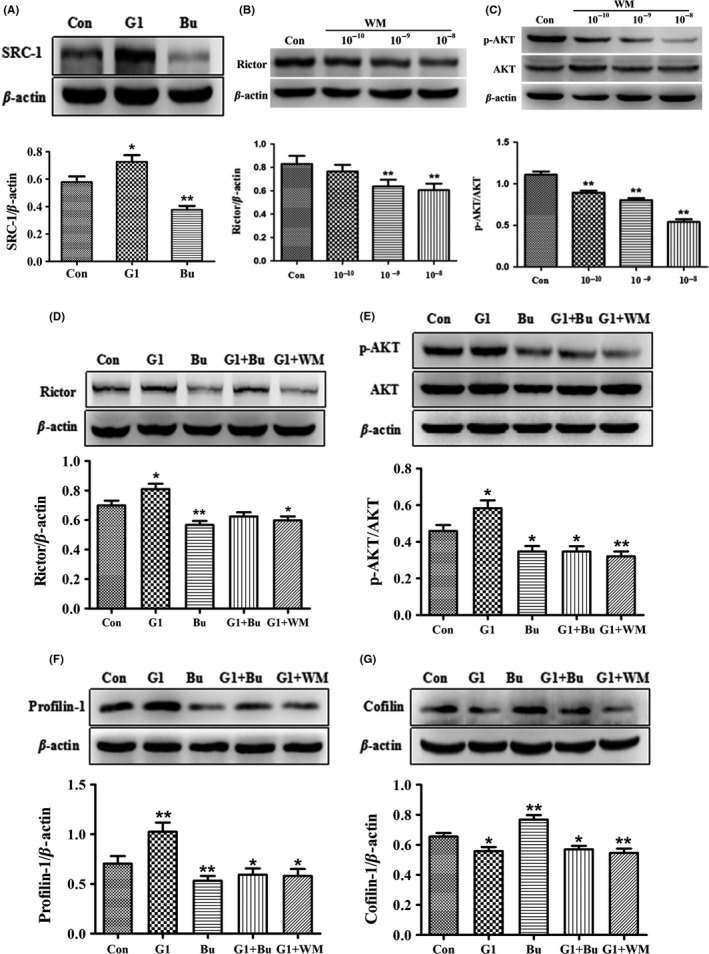
SRC‐1 and PI3K inhibition affected G1‐induced mTORC2 signals and actin polymerization remodeling proteins in vitro. A, SRC‐1 was upregulated by G1 but inhibited by Bufalin. B‐C, Levels of Rictor and p‐AKT were dose‐dependently regulated by Wortmannin. D‐E, G1‐induced increases in Rictor and p‐AKT were inhibited by SRC‐1 and PI3K inhibition. F, G1‐induced increase in Profilin‐1 was inhibited by SRC‐1 and PI3K inhibition. G, G1‐induced decrease in Cofilin was inhibited by SRC‐1 and PI3K inhibition. Bu: SRC‐1 antagonist Bufalin. WM, PI3K antagonist Wortmannin; Con, control (DMSO treated). The experiments were repeated three times, and data are shown as the mean ± SEM *P < 0.05, **P < 0.01 compared with the Con (one‐way ANOVA, LSD test)

**Figure 8:**
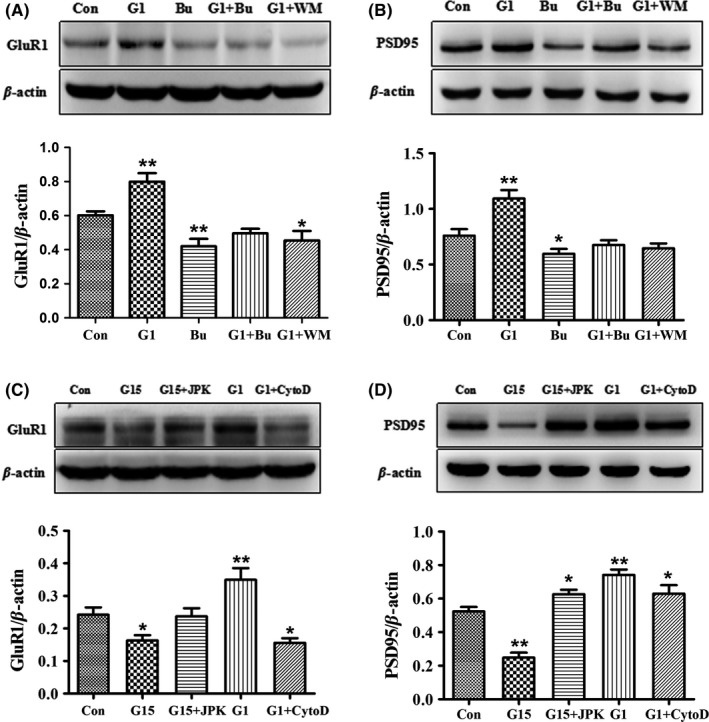
GPR30 activity modulation‐induced changes in synaptic proteins were blocked by inhibition of SRC‐1, PI3K, and regulation of actin polymerization in vitro. A‐B, G1‐induced increases in GluR1 and PSD95 were inhibited by SRC‐1 or PI3K inhibition. C‐D, G15‐induced decreases in GluR1 and PSD95 were reversed by induction of actin polymerization with JPK; G1‐induced increases in these two proteins were blocked by disruption of actin polymerization with CytoD. Bu, SRC‐1 antagonist Bufalin; WM, PI3K antagonist Wortmannin; CytoD, cytochalasin D; JPK, jasplakinolide; Con, control (DMSO treatment). The experiments were repeated three times, and data are shown as the mean ± SEM *P < 0.05, **P < 0.01 compared with the Con (one‐way ANOVA, LSD test)

Bufalin has been shown to inhibit SRC‐1 in cancer cells,^43^ and Wortmannin is an effective PI3K inhibitor.^51^ To explore whether SRC‐1 and PI3K were involved in the GPR30 regulation of mTORC2 signals, actin cytoskeleton remodeling, and synaptic proteins, we treated the mHippoE‐14 cells with G1, Bufalin, and Wortmannin separately or in combination. We first demonstrated that the levels of SRC‐1 were upregulated by G1 and inhibited by Bufalin; one‐way ANOVA and the LSD test showed that the expression of SRC‐1 was significantly different among the control, G1, and Bufalin (*F*(2,15) = 19.339, *P* < 0.01). SRC‐1 was significantly inhibited by 35% after Bufalin treatment and increased by 26% after G1 treatment (Figure 7A: *P* < 0.05 compared with Bufalin; *P* < 0.01 compared with G1). For the concentration of Wortmannin, one dose‐dependent preliminary test regarding its effects on hippocampal Rictor and p‐AKT was conducted (Figure 7B‐C) and 10^−8^ mol/L was used for subsequent experiments. For mTORC2 signals, the results showed that their expression was significantly upregulated by G1 and downregulated by Bufalin or Wortmannin as shown in Figure 7D‐E. The Bufalin‐induced decreases in these proteins were reversed to the control level by combined Bufalin and G1 treatment, and the G1‐induced increases in these proteins were decreased again by Wortmannin (Figure 7D: *F*(4,25) = 10.775, *P* < 0.01 for Rictor; Figure 7E: *F*(4,25) = 11.204, *P* < 0.01 for p‐AKT). For the expression of profilin‐1 (*F*(4,25) = 11.599, *P* < 0.01 among group), the results showed that its expression was significantly upregulated by G1 and downregulated by Bufalin or Wortmannin (Figure 7F). For the expression of Cofilin (*F*(4,25) = 12.038, *P* < 0.01 among groups), it was significantly downregulated by G1 or Wortmannin and rescued by Bufalin (Figure 7G). For the expression of the selected synaptic proteins GluR1 and PSD95 (*F*(4,25) = 13.488, *P* < 0.01 for GluR1; *F*(4,25) = 13.412, *P* < 0.01 for PSD95), their expressions were upregulated by G1; however, this effect was significantly inhibited by Bufalin or Wortmannin as shown in Figure 8A‐B.

### 3.8 GPR30 activation regulated synaptic proteins through actin polymerization

Previous studies have shown that microtubule inhibition decreased the expression of PSD95^52^ and actin polymerization affected the number of GluR1‐positive spines,^53^ which indicates a close relationship between cytoskeleton dynamics and these synaptic proteins. JPK and CytoD have been used to regulate actin polymerization in a previous study.^37^ We employed these two compounds to treat mHippoE‐14 cells in combination with G1 or G15. One‐way ANOVA analysis showed that there were significant differences among the five groups (*F*(4,25) = 10.376, *P* < 0.01 for GluR1; *F*(4,25) = 29.299, *P* < 0.01 for PSD95). The G15‐induced decrease in PSD95 and GluR1 was significantly reversed by JPK, and the G1‐induced increase in these two proteins was significantly inhibited by CytoD as shown in Figure 8C‐D.

### 3.9 GPR30 inactivation‐induced spatial memory impairment and actin depolymerization were restored by mTORC2 activation

**Figure 9:**
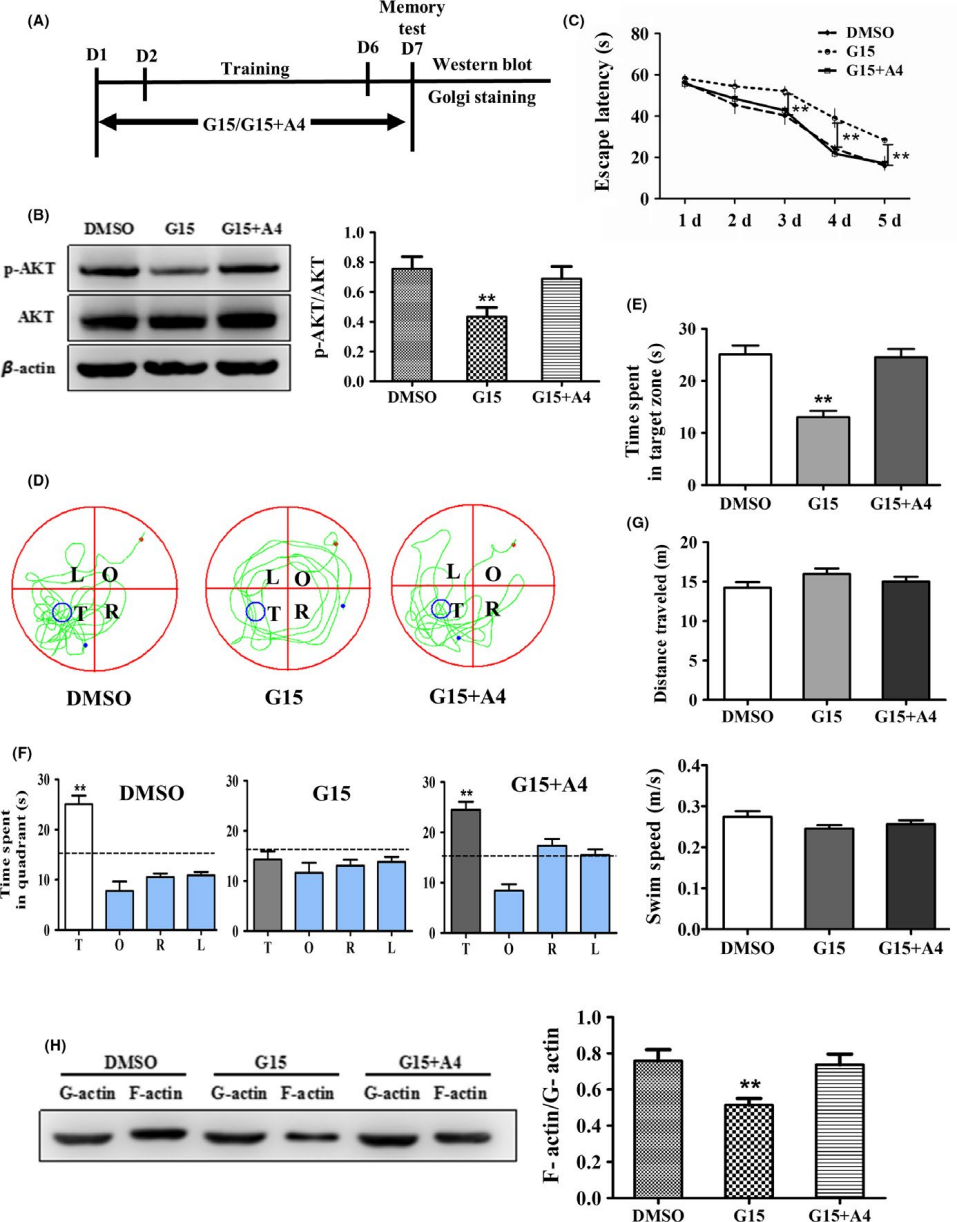
Selective activation of mTORC2 reversed G15‐induced spatial memory disorder and actin depolymerization. The sham animals were in diestrous cycling (evidenced by vaginal smear). The mice injected with DMSO were used as the control. A, Flowchart of the experiments. B, A4 reversed G15‐induced learning impairment from days 3 to day 5. C, The swimming tracks of mice in each quadrant. D, The time spent in the target quadrant of animals. E‐G, The total distance traveled and swimming speed. H, A4 rescued G15‐induced downregulation of p‐AKT. I, A4 reversed G15‐induced decrease in F‐actin/G‐actin ratio. A4: mTORC2 activator A‐443654. Data are shown as the mean ± SEM. **P < 0.01 compared with other groups (repeated measures of two‐way ANOVA, LSD test for water maze test and one‐way ANOVA, LSD test for Western blot analysis)

A4 has been shown to induce rapid AKT Ser‐473 phosphorylation^54^ and thus activate mTORC2 and promote actin polymerization.^37^, ^55^ To determine whether GPR30 regulation of learning and memory is mTORC2‐dependent, we used A4‐ to G15‐treated mice under the strategy shown in Figure 9A. The Morris water maze test subsequently indicated that during the 5 days, the escape latency was significantly affected by both day (*F* = 87.882, *P* < 0.01) and treatment (*F* = 20.923, *P* < 0.01); however, there was no detectable day × treatment interaction (*F* = 1.287, *P* > 0.05, repeated two‐way ANOVA, LSD test) (Figure 9B‐E). G15 or G15 + A4 did not affect the swimming distance and speed of the animals (Figure 9F: *F*(2,15) = 1.701, *P* > 0.05 for distance; Figure 9G: *F*(2,15) = 1.740, *P* > 0.05 for swimming speed). The results demonstrated that the G15‐induced decrease in p‐AKT was restored by A4 administration as shown in Figure 9H. Moreover, one‐way ANOVA analysis showed that F‐actin/G‐actin was significantly different among the DMSO, G15, and G15 + A4 treatments (*F* = 6.344, *P* < 0.01). The G‐15‐induced decrease in F‐actin/G‐actin was also significantly restored by A4 as shown in Figure 9I.

## 4 DISCUSSION

### 4.1 Postnatal and estrogenic regulation of hippocampal GPR30

Estrogens have been shown to be important regulators of memory and cognition through action on hippocampal synaptic plasticity that depends on actin cytoskeleton remodeling. Although estrogen replacement therapy has been shown to be beneficial for AD patients, it also increases the risk of hormone‐sensitive cancers, such as breast cancer.^56^ Therefore, it is important to explore the molecular mechanisms that underlie estrogenic action to provide novel targets for the prevention and treatment of AD, as well as other estrogen deficiency‐related neural disorders.

Previous studies have indicated high levels of GPR30 in the hippocampus of adult male and/or female rats^21^, ^57^, ^58^; however, the exact role of this ER and related mechanisms in the hippocampus remain unclear. To this end, we initially examined the expression of GPR30 in the hippocampus of postnatal female mice. The results showed that in the early postnatal days, the levels of GRP30 were relatively low and subsequently increased to the highest levels at P56, which indicates an increased profile of GPR30 in the hippocampus. These results were in agreement with previous results that indicated high levels of GPR30 in the hippocampus of male and female rats.^21^, ^57^, ^58^ As aging induces a decrease in estrogens derived from both circulating and local synthesis, we used OVX and LET to mimic these changes and determined that both treatments induced significant decreases in hippocampal GPR30. These results strongly indicated that hippocampal GPR30 may mediate estrogenic regulation of hippocampal structure and function.

### 4.2 Pharmacological modulation of GPR30 activity affects spatial memory

To explore the function of the GPR30 in the hippocampus, the GPR30 activity was pharmacologically manipulated using its specific agonist or antagonist to explore the mechanisms that underlie GPR30 in the estrogenic regulation of spatial memory. The GPR30 agonist G1 has been used to investigate the role of the GPR30 in the regulation of learning and memory, as well as cognition in female rats^24^, ^25^ and the social recognition, object recognition, and object placement learning of female mice.^28^, ^30^ However, studies have rarely reported the effects of the GPR30 antagonist G15 treatment on learning and memory, although it has been shown that G15 inhibited E2‐induced hippocampal synaptic transmission in adult female mice,^59^ as well as AKT phosphorylation in adult female rats.^60^ In this study, we used both G15 and G1 to treat animals and examined their effects on learning and memory using the Morris water maze. The results showed that after G15 treatment, animals exhibited significantly longer escape latency during the learning phase and a shorter time in the target zone during the memory test phase. Furthermore, we determined that OVX induced longer escape latency and a shorter time in the target zone; these effects were significantly reversed by G1 treatment. Under both conditions, the swimming speed and total swimming distance traveled were not affected. These results were in accordance with previous studies that showed GPR30 activation by G1 rapidly enhanced learning in female CD1 mice^61^ and ameliorated long‐term recognition memory in an AD mouse model (5XFAD) of male and female mice^62^ and G15 impaired the acquisition of a spatial learning task in young female rats,^26^ thus suggesting the important role of GPR30 in mediating the estrogenic effects on spatial learning and memory.

### 4.3 Pharmacological modulation of GPR30 activity affects actin polymerization and synaptic proteins

Actin cytoskeleton dynamics have been shown to control the structural and functional plasticity of dendritic spines and ultimately affect learning and memory.^63^, ^64^ To investigate the underlying mechanism of GPR30 regulation on learning and memory, we initially examined the changes in the dendritic spine density, F‐actin/G‐actin ratio, and levels of Profilin‐1 and Cofilin after G15, OVX, and/or G1 treatment. Our in vivo and in vitro evidence demonstrated that G15 and OVX significantly decreased the spine density, F‐actin/G‐actin ratio, and Profilin‐1, which were increased by G1; moreover, the expression of Cofilin was significantly increased by OVX or G15 and decreased by G1. These results strongly indicated a pivotal role of GPR30 in the regulation of the dendritic spine density through regulating actin polymerization. However, to date, rare studies have reported the effects of GPR30 on the actin cytoskeleton polymerization dynamics. Carnesecchi et al^65^ reported that a lack of E2 induced rapid re‐organization of the human dermal fibroblast cytoskeleton, which resulted in striking cell shape changes that were completely restored by E2 treatment; these effects were mimicked by the GPR30 agonist G1 and the antagonist G15. Gabor et al^30^ reported that GPR30 activation by G1 increased the CA1 dendritic spine density of female mice, and Xing et al^38^ indicated that G15 injection could induce actin depolymerization in the hippocampus of female mice. Thus, our current results provide additional evidence regarding GPR30 and the hippocampal dendritic spine density. PSD95 is one of the most important synaptic scaffold and hub proteins enriched at glutamatergic synapses and is involved in aging, AD, and numerous psychiatric disorders.^66^ GluR1 (also referred to as GluR‐A) is an AMPA‐type glutamate receptor, which has been shown to be important for learning and memory because of its role in long‐term potentiation induction and synaptic plasticity.^67^ GPR30 has been detected in the dendritic shafts, synaptic specializations in hippocampal dendritic spines of both male and female mice,^23^ particularly the postsynaptic density.^68^ Occasional studies have reported that GPR30 activation increased the expression of PSD95 in the CA3 of the hippocampus of male and female mice^23^ and the clustering of the GluR1 to excitatory synapses as shown in the spinal cord of female rats.^69^ In this study, we determined that the expressions of PSD95 and GluR1 were significantly upregulated by G1 and downregulated by G15 and these G15 effects were similar to our previous findings in adult female mice,^38^ which indicates GPR30 functions to regulate synaptic plasticity, at least in part, by acting on these synaptic proteins.

### 4.4 SRC‐1 and PI3K mediate GPR30 regulation on actin polymerization and synaptic proteins

To investigate the potential interaction between GPR30 and the classic nuclear estrogen receptors (ERα and ERβ; which have been detected in the cell membrane and cytoplasm), we examined the expression of ERα and ERβ, as well as their coactivator SRC‐1, which is highly expressed in the hippocampus and regulated by aromatase inhibition and aging in our previous studies using adult female rats.^48^, ^70^ The in vivo results showed that both G1 and G15 treatments induced significant changes in nuclear ERs and SRC‐1, which indicates the interaction between the classic nuclear ERs and the novel membrane ER GPR30 as reviewed by Kosaka et al^71^; the in vitro studies showed the E2‐induced increase in SRC‐1 was significantly inhibited by the GPR30 antagonist G15, which indicates SRC‐1 is also the downstream target of GPR30. To extend this finding, we employed Bufalin, a specific antagonist against SRC‐1,^43^ to inhibit SRC‐1 and subsequently examined the expression of actin polymerization‐regulating proteins under G1 treatment. We determined that when the cultured hippocampal cells were treated with G1 and/or Bufalin, the levels of the G1‐induced increase in Profilin‐1 were significantly decreased, whereas the expression of the G1‐induced decrease in Cofilin was significantly increased, which indicates SRC‐1 also mediated GPR30 regulation on actin cytoskeleton polymerization. Furthermore, we determined that the levels of PSD95 and GluR1 were significantly decreased when a G1 combined treatment with SRC‐1 inhibition strategy was used, which indicates SRC‐1 also mediated GPR30 regulation on these two proteins. Conclusively, these results indicated the existence of classic nuclear ER (ERα and ERβ)‐novel membrane ER (GPR30/GPR30) interaction and SRC‐1 could also mediate the effects of GPR30 on hippocampal synaptic plasticity and thus affect learning and memory.

The PI3K signaling pathway has been shown to be involved in the effects of GPR30. For example, activation of GPR30 by G1 shows an anti‐apoptotic effect through PI3K following spinal cord injury in adult female mice^72^, ^73^; moreover, inactivation of GPR30 by G15 inhibits E2‐induced activation of PI3K in Boar Sertoli cells^74^ and AKT phosphorylation in endometrial cancer cells.^75^ Furthermore, GPR30 has been shown to regulate neurite genesis in developing hippocampal neurons via PI3K activation.^51^ However, it is unclear whether PI3K mediates GPR30 regulation of actin cytoskeleton polymerization and synaptic proteins. We found that in cultured neurons, G1 induced increases in the actin regulatory protein Profilin‐1, synaptic protein GluR1, and PSD95 and the decrease in the actin polymerization disruptor Cofilin was prevented by the PI3K inhibitor Wortmannin, which indicates the important role of PI3K in the mediation of GPR30 regulation on these synaptic plasticity‐related proteins.

### 4.5 Actin polymerization mediates GPR30 regulation of synaptic proteins

It has been reported that in mouse hippocampal neurons, the brain‐derived neurotrophic factor‐induced increase in PSD95 was abolished by microtubule inhibition^52^ and depolymerization of F‐actin could induce a decrease in GluR1,^53^ which indicates these two proteins could be regulated by cytoskeleton plasticity. However, whether GPR30‐induced actin polymerization also affects the expression of GluR1 and PSD95 remains unclear. In this study, we determined that G15 or G1 treatment significantly affected the levels of hippocampal GluR1 and PSD95, E2‐induced increase in GluR1 and PSD95 was significantly inhibited by G15. We subsequently demonstrated the G15‐induced decrease in PSD95 and GluR1 was reversed by promoting actin polymerization with JPK, and the G1‐induced increase in PSD95 and GluR1 was inhibited by the disruption of actin polymerization with CytoD. These results were consistent with a previous report by Allison et al^53^ that showed the levels of GluR1 were regulated by actin polymerization, which indicates actin cytoskeleton dynamics also profoundly affect the expression of synaptic proteins and ultimately the synaptic strength.

### 4.6 mTORC2 integrates signals from SRC‐1 and PI3K, and mTORC2 activation rescues G15‐induced actin depolymerization and memory impairment

Rictor is the defining component of mTORC2 and is required for mTORC2 stability and function as shown in male mice^37^; AKT phosphorylation at Ser‐473 has been shown to be the best characterized readout of mTORC2 activity.^76^ A previous study by Huang et al^37^ demonstrated that Rictor deletion induced actin depolymerization (as shown by F‐actin/G‐actin) and impaired memory, which could be restored by actin polymerization induction as detected in male mice. However, how mTORC2 functions in the mediation of GPR30 regulation of actin polymerization and spatial learning remains unclear.

In this study, we first determined that the OVX‐induced decrease in Rictor and p‐AKT was significantly reversed by GPR30 activation with G1, and the expression of these two proteins was significantly decreased by GPR30 inactivation with G15. These findings were further supported by in vitro studies that showed E2‐induced Rictor and p‐AKT were inhibited by G15. Furthermore, we determined that when SRC‐1 or PI3K was inhibited, the levels of Rictor and p‐AKT were also significantly downregulated, which indicated mTORC2 is the downstream target of SRC‐1 and PI3K. Finally, we demonstrated that G15‐induced actin depolymerization and spatial memory disorder were significantly reversed by activating mTORC2 with the mTORC2 activator A4. Limited studies have shown that direct activation of mTORC2 could affect actin polymerization. For example, Johnson et al^77^ showed that in the brains of both fruit flies and rodents, the activity of mTORC2 declined with age and the loss of mTORC2‐mediated actin polymerization contributed to age‐associated memory loss, which could be reversed by A4‐directed mTORC2 activation. Sun et al^55^ reported that in the hippocampal slices of male mice, mTORC2 activation by A4 increased hippocampal actin polymerization. However, to our knowledge, the current study is the first report regarding the role of mTORC2 in the regulation of GPR30 on actin polymerization, as well as learning and memory; these results strongly indicate the pivotal role of mTORC2 in GPR30‐guided structural and functional plasticity.

Aging‐induced neurodegenerative diseases, such as AD, deeply affect the life quality of humans. Estrogens, from circulating or local synthesis in the brain, have been shown to play important roles in this neurodegenerative process. In this study, we demonstrated that GPR30 regulated learning and memory through ERα/ERβ/SRC‐1 and PI3K, subsequent mTORC2 activation and downstream actin cytoskeleton polymerization which regulated the expression of synaptic proteins. Admittedly, the current findings in this study are very preliminary which need further experiments such as RNA interference and/or gene knockout against GPR30 for verification. However, these results still provide some novel insights into understanding the role that GPR30 plays in the estrogenic action on the pathology of AD and they also indicate this receptor may represent one preventing and therapeutic target against hippocampus‐based, estrogen‐related learning and memory impairments.

## CONFLICT OF INTEREST

The authors declare that they have no conflicts of interest.
