# Supplementary figures and images for: GPR30‐mediated estrogenic regulation of actin polymerization and spatial memory involves SRC‐1 and PI3K‐mTORC2 in the hippocampus of female mice
Source: CNS Neurosci Ther. 2019 Feb 3;25(6):714–33. doi: 10.1111/cns.13108 (PMC6515707; doi:10.1111/cns.13108)

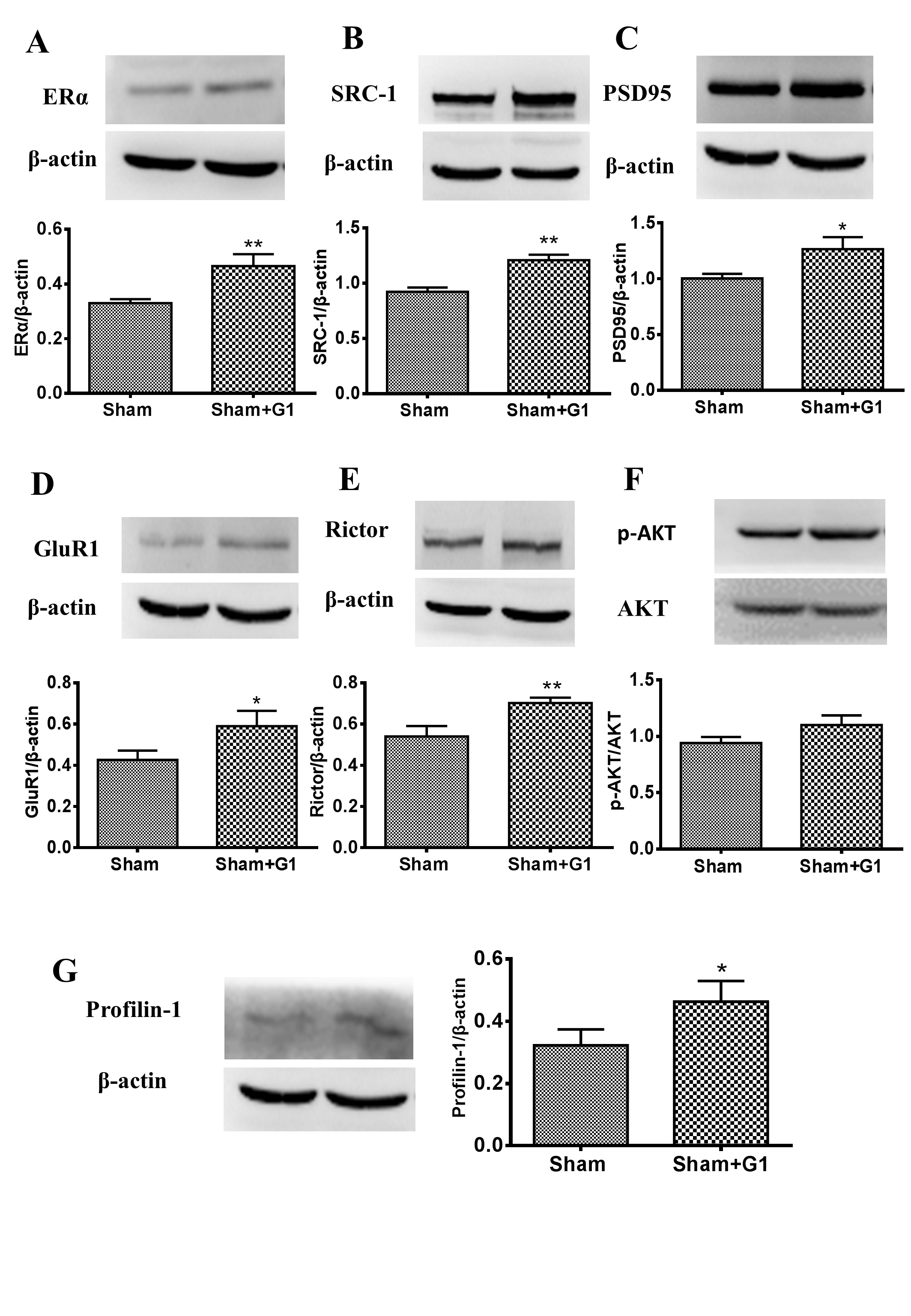

Supplement: Supplementary file 1 [file CNS-25-714-s001.tif]
